# Supplementary material for: Practice of and associated factors regarding prevention of surgical site infection among nurses working in the surgical units of public hospitals in Addis Ababa city, Ethiopia: A cross-sectional study
Source: PLoS One. 2020 Apr 16;15(4):e0231270. doi: 10.1371/journal.pone.0231270 (PMC7161981; doi:10.1371/journal.pone.0231270)
Supplement: Attachment — Submitted filename: Response to reviewers.docx [file pone.0231270.s002.docx]

**Response to Reviewers**

Dear reviewer(s)

Greetings,

First of all I thank you very much on behalf of the co-authors for your genuine and constructive comments you raised while reviewing the article. Therefore, based on the comments raised, I have tried to clarify some points and modifying some others which need modification and correction. So, I put the clarifications and corrections made during revision as follows.

Thank you very much!

Yours sincerely,

Principal author

**Point to point response**

- ***General corrections made include: rephrasing of the title, grammar and spelling corrections, grammar and punctuation marks with grammerly proofreading, general formatting, alignment of texts in the tables, deletion of % in the tables etc. The validity and reliability issues are well described.***

***Details on reviewer comments;***

1. Please include a copy of the survey questions or questionnaire used in the study, in both the original language and English, as Supporting Information, or include a citation if it has been published previously. If the original language is written in non-Latin characters, for example Amharic, Chinese, or Korean, please use a file format that ensures these characters are visible.

*Here is the English version questionnaire which was used to measure nurse’s practice, because English is an academic language in Ethiopia and it has been cited.*

### Annex II: Structured English Version Questionnaire

**Section One: Questions on Socio Demographic Characteristics of Participants**

Please read and encircle the number that belongs to you and write your answer on the blank spaces for the questions asking you to write your answer.

| **S. No** | **Questions** | **Responses** |
| --- | --- | --- |
| **101** | Age | __________years |
| **102** | Sex | 1. Male 2. Female |
| **103** | Marital status | 1. Single 2. Married 3. Divorced   4. Widowed 5. Separated |
| **104** | Educational status | 1. Diploma 2. Degree 3. Masters |
| **105** | Total monthly income | ___________Ethiopian birr |
| **106** | Total work experience | 1. 0-5 years 2. 6-10 years 3. 11-15 years   4. 16-20 years 5. Above 20 years |
| **107** | Work experience in the surgical units | ­­­­­­­­­­­­­­­­­­­­­­­­­__________ years |
| **108** | Name of duty ward | 1. Surgical 2. Orthopedics 3. Recovery   4. Gyn. and labor 5. Others (specify)________ |
| **109** | Level of the hospital you are working in? | 1. Primary hospital 2. Secondary hospital 3. Tertiary hospital |
| **110** | Have you ever taken training regarding infection control? If no, go to question number **112**. | 1. Yes 2. No |
| **111** | If yes for the above question, mention the number of infection control trainings you attend. | ­­­­­­­­­­­­­­­­­­­­­­  _________Times |
| **112** | Is there a surgical site infection prevention guideline in your hospital? If no, go to section two. | 1. Yes 2. No |
| **113** | If yes for question number 112;  Are you using it? | 1. Yes 2. No |

**Section Two**: **Questions Used To Assess Nurses level of Practice Regarding Prevention of Surgical Site Infection:**

Please answer the following questions by giving the mark (**X**) on the column that best fit to your current level of practice on each item while you were on the situation honestly.

| **Sr.No.** | **Questions** | **Never** | **Seldom** | **Sometimes** | **Always** |
| --- | --- | --- | --- | --- | --- |
| **301** | How often do you use alcohol and chlorhexidine gluconate as an antimicrobial in your ward? |  |  |  |  |
| **302** | How often do you wash your hands before and after changing wound dressings or touching surgical sites? |  |  |  |  |
| **303** | How often do you wash your hands before wearing surgical gloves? |  |  |  |  |
| **304** | How often do you perform preoperative shaving on the day before surgery? |  |  |  |  |
| **305** | How often do you administer preoperative prophylactic antibiotics within 120 minutes, but, not later than30 minutes before surgery? |  |  |  |  |
| **306** | How often do you learn shaving methods from others? |  |  |  |  |
| **307** | How often do you advise patients to take pre-operative showering within 6-12 hours before surgery? |  |  |  |  |
| **308** | How often do you advise patients to take pre-operative showering with antimicrobial agent? |  |  |  |  |
| **309** | How often do you perform prescribed glucose test before and after surgery in a diabetic patient? |  |  |  |  |
| **310** | How often do you assess the patient’s body mass index (BMI) before and after surgery? |  |  |  |  |
| **311** | How often do you administer injection insulin or give oral medication as ordered in diabetic patient? |  |  |  |  |
| **312** | How often do you advise obese patients to reduce the amount of carbohydrate they take? |  |  |  |  |
| **313** | How often do you advise a malnourished patient to take nutritious diet (especially protein diet)? |  |  |  |  |
| **314** | How often do you advise a patient with a compromised immune system to avoid contact with people who have infections? |  |  |  |  |
| **315** | How often do you advise a malnourished patient to take vegetables and fruits before & after surgery? |  |  |  |  |
| **316** | How often do you use sterile forceps and/or sterile dressing materials for cleaning and dressing of surgical wound? |  |  |  |  |
| **317** | How often do you advice patients with immunodeficiency disorder to maintain their personal hygiene? |  |  |  |  |
| **318** | How often do you follow an aseptic technique during obtaining swab culture? |  |  |  |  |
| **319** | How often do you follow an aseptic technique during surgical wound dressing? |  |  |  |  |
| **320** | How often do you use povidone-iodine and normal saline for cleansing surgical wound dressing? |  |  |  |  |
| **321** | How often do you asses and monitor surgical site conditions? |  |  |  |  |
| **322** | How often do you separate infected dressing from non-infected dressing? |  |  |  |  |
| **323** | How often do you wear face mask during cleaning surgical wound and change dressings? |  |  |  |  |
| **324** | How often do you perform cleaning and disinfect surface of the dressing trolley with anti-septic solutions? |  |  |  |  |
| **325** | How often do you discard the soiled materials in the proper place after performing wound dressing? |  |  |  |  |
| **Subsection: Answer the questions accordingly.** | | | | | |
| **326** | How do you rate the overall level of your current practice regarding prevention of surgical site infection? Encircle your choice.   1. Very unsatisfactory 3. Satisfactory 2. Unsatisfactory 4. Very satisfactory | | | | |
| **327** | If you are not very satisfied with your practice, what are the reasons? **Note:** More than one answer is possible. Encircle your choice.   1. I have no sufficient knowledge about surgical site infection prevention 2. Inadequate resources to implement surgical safety checklists in the hospital 3. Insufficient performance monitoring systems related to surgical site infection prevention 4. Lack of surgical site infection assessment and preventive measure feedback systems 5. Others:________________________________________________________________________________________________________ | | | | |

2. Please state whether you validated the questionnaire prior to testing on study participants. Please provide details regarding the validation group within the methods section. ***Yes*** *it has been validated and has been stated in the main document under data quality assurance section*. **It reads as,** to ensure the quality of data, the questionnaire was pretested on 5% (21) of the sample size. The pretest study was conducted two weeks before the actual data collection period at Zewditu memorial hospital. The hospital was selected by lottery method among the hospitals which weren’t included in the study settings. The study subjects who fulfill the inclusion criteria were selected by simple random sampling technique. In reliability test, a Cronbach’s Alpha coefficient of 0.89 was obtained

**Reviewer #1:**

1. Title: seems good if “Surgical Site infection prevention practice and associated factors among Nurses in Public Hospitals in Addis Ababa City, Ethiopia, 2018”,. *The title has been revised and shortened.*

1.1. Capitalization for all words is not recommended. *Corrected and only the first letter is capitalized.*
2. Abstract:
2.1. Introduction: it is good to explain “why the study is needed” *explained;*

2.2. Method: What was the strata?, better to describe. *Described, the strata were the level of hospitals nurses are working in and were stratified as tertiary and secondary level hospitals. Because, there was no primary level hospital in Addis Ababa city*

3. Introduction: Grammatical errors are common, especially in the 1st and 2nd paragraphs *grammatical errors are corrected using grammerly.*

4. Methods and Materials:
4.1. Study period: I understand that the time mentioned is data collection period. ***Of course yes. Study period is the period in which actual data is collected.***
4.2. Sample size: the proportion used (48.7%) was done 3 years ago, in the same country and it is similar result with the current findings. Within this period, it is not expected to found contextual difference. Could you clearly justify why this study is needed?

*This study was conducted to assess the practice of nurses regarding surgical site infection in the three levels of hospitals in Addis Ababa city and to identify the possible factors, especially institution related factors which can affect the nursing practice among the different levels of the hospitals unlike the previous study which was conducted only among tertiary hospitals in one region of the country. In addition this study only involved nurses working in surgical units unlike the previous one which included all nurses in the hospitals it included, and we believe that, there are nurses who never work in surgical units and asking them about their practice regarding prevention of SSI is most probably a source of bias. Because asking a person about how he or she is performing an activity that he is not expected to perform seems illogical. For example if the data collection method for the previous study was direct observation, were they going to observe the practice of nurses regarding SSI prevention who actually weren’t working in the surgical related units?. It seems inappropriate. Because of the two major reasons we decided to conduct the study. Unfortunately, the results of the two studies were close to each other.*

4.3. Sampling technique: There is no difference among the first list of sites and the finally selected sites in social and contextual values. What is the importance of the design effect? I suggest not to use.

*This was in fact a source of argument among the researchers and finally we decided as long as we stratified the hospitals as secondary and tertiary in level, we will already bypass some of the hospitals during the selection time. So it is better to use design effect and increase the sample size so as to minimize error. Because, we believe that the level of hospitals nurses are working in can have a huge effect for differences in nurses practice level in terms of providing an opportunity for training, formal educational chance etc. Therefore, our decision was based on this assumption.*

4.4. Data Collection Technique and Tools: it is understood that outcome is measured by the degree of practice that the respondents said. There may be number of activities expected to confirm just to conclude their practice. Because this paper I think measures their level of thinning or believe that they are practicing SSI prevention; therefore this could be better to describe as “Attitude” than practice.

*Of course the checklist was used to assess how often they were practicing some of the critical SSI prevention activities which they are expected to always practice properly. So, we thought asking them how often they were practicing these activities properly during their work directly measures their practice rather than their attitude. In fact attitude and practice are inseparable, because we practice based on what we believe. Similarly, the checklist was used in the same manner by other researchers and has been cited.*

4.4.1. The questionnaire is pretested, but three days before data collection, training was given. When was done the pretest?

*The pretest study was conducted* ***2 weeks before the actual data collection period*** *at Zewditu memorial hospital which was not included in the study settings and was selected by lottery method among the hospitals not included in the study settings. The study subjects who fulfill the inclusion criteria were selected by simple random sampling technique. The data for pretest was collected by investigators, not by data collectors.*

5. Results:
5.1. “Only 224 (54.8%) of the study participants claim they have taken training regarding infection control methods (Table 1). = “only” seems small number, but it is > 50%.

All of those who work in surgical units are expected to have the necessary knowledge and skills on SSI Prevention methods. To have that, they should take training. We believe 54.8% is not enough and if possible all of them should take training on SSI infection prevention methods. But, as a general rule we have accepted the comment and made corrections.

5.2. Table 1: age is categorized in to two, what is the reason? *This was based on previous researches, so as to make it comparable we followed similar approach.*

5.3. Nurse’s Practice Regarding Prevention of Surgical Site Infections: repetition is better to remove, example: (the score ranging from 0 to 75). *Corrected.*

5.4. Factors Associated with Nurse’s practice regarding Prevention of Surgical Site Infections: what do you mean by “Training had a week association with practice at a p value of 0.05”

*As far as, a p value of <0.5 is considered as a cut of point to declare statistically significant value. We believe that, this has to be corrected and we accepted and corrected it*.

5.5. “Nurses were asked to rate their level of practice, and 56 (13.7%) of them rated as unsatisfactory”. Based on this statement, if this is qualitative study including in your method; otherwise it is confusing with the main outcome which was measured using Likert scale. What is their difference?

*After they finished filling the Liker scale, they were asked to rate the overall performance of their practice and an open ended question to mention the possible factors which were influencing their practice. So, their personal rating of their overall practice does not influence the outcome that is measured using the Likert scale. Secondly, even though the study was primarily quantitative, the intention here was to explore all the possible factors which were influencing their practices so that to give an impression for future researchers to study all the possible variables as much as possible.*

6. Discussion:
6.1. The second paragraph looks like recommendation. *Modified*

6.2. ‘Mean practice score being 77.8%”, what do you mean by this statement, because mean score is not proportion. *It has been already explained and defined in result part as mean =* 58.36 (77.8%))*. So, corrected accordingly.*
6.3. Properly or correct practice is measured by observation, but in your discussion “improper practice is used incorrectly.

As we explained above, the checklist was used to measure whether they were following *critical procedures strictly or not during their practice. So, even though the data collection technique was not observational, the checklist directly measures properness of nurse’s practice regarding surgical site infection prevention*. *But, we also share the point raised that, direct observation is one of the best methods which avoids* central tendency, acquiescence and social desirability biases with this regard. Otherwise as long as the checklist was designed to measure their practice whether it was proper or not proper and adequate information was provided to study participants to provide honest information regarding their actual practice, we believe it will not matter here.

6.4. Paragraph 4: “But the result is in contrast with studies in Pakistan and another study in Bangladesh in that the overall practice of staff nurses regarding preventing and managing surgical site infection was at good level (20, 21). This seems meaningless, may need rewriting. *Modified*

6.5. Paragraph 5: the justification is given for the association, but not for the difference or similarity of findings. The justification is against the finding. *Modified accordingly*

7. Conclusion: “almost half of the participants” is different from “more than half” *Modified*.
7.1. Paragraph 1: In sufficient knowledge, inadequate resources to implement surgical safety checklists, insufficient performance monitoring systems, lack of surgical site infection assessment and preventive measure feedback systems, and others, like; excessive workload, staff inadequacy, lack of training, insufficient orientation programs during unit rotation and harassing hospital environment were also identified as factors affecting the nurse’s practice regarding prevention of SSIs. Per my understanding, this is not identified as factors affecting the practice. Rather it is mentioned by participants, may be qualitative expression. Could you confirm that variables have association? What ware the direction and strength and association?

*As we said above, after they finished filling the Liker scale, they were asked to rate the overall performance of their practice and an open ended question to mention the possible factors which were influencing their practice. So, their personal rating of their overall practice does not influence the outcome that is measured by using the Likert scale. Secondly, Even though the study was primarily quantitative, the intention here was to explore all the possible factors which were influencing their practice. This was simply to present an impression on the many possible factors for future researchers. Otherwise no associative analysis was done on the factors which were mentioned by nurses. Associative analysis was done only for the variables that are present in the table under the manuscript.*

Final conclusion:
I do have double on the understanding of the measurement of outcome variable.
Practice it better to be measured using “activities” whether done or not done in their working experience. The way that this paper is used to measure seems rather “Attitude”
Therefore this might need Major correction, since outcome is the center of discussion and the study does not presents the results of primary outcome.

*As we explained above the checklist was used to assess how often they were practicing (following strict procedures) some of the critical SSI prevention activities which they are expected to practice/follow always during the pre operative, intra-operative and post operative phases. So, asking them how often they were practicing these activities properly during their work directly measures their practice weather it was proper or not than their attitude even though practice and attitude are almost always inseparable. Similarly, the checklist was used in the same manner by other researchers.*

*We have found more than 7articles from different data bases which used the same checklist for the same outcome. But, we also share that direct observation is one of the best method to measure the properness of practice.*

*Thank you very much!*

*We have considered all the genuine points you raised and made necessary corrections.*

**Reviewer #2:** Dear author(s),
 Congrats on your research done and this well-written manuscript. Below are some comments for better improvement:
1) Abstract - please write the study duration. *Written*
2) Introduction – okay
3) Methods
-Study area, design and period....typo...”from March 01 to 30, 2018”. *Corrected*
-Data collection technique and tools – please explain further regarding the tool – e.g., adapted from? Previous studies? Please make proper citation. *Cited*
-Data quality assurance – please explain the reliability analysis as this tool was adapted from other sources (e.g., Cronbach's alpha). *Modified*
-Data processing and analysis – please check again as you did not presented in your graphs (as stated in your manuscript; last paragraph. *Seen* and *modified*

4) Results
-Table 1 – “duty ward” – "Others" (please give examples). *Given*
-Nurse’s practice regarding prevention of surgical site infections – 1st paragraph, it would be better if you can provide a Table to explain the section. *Given below the next paragraph*
-Table 2 – you may re-arrange the Table as you not able to discuss all the items in the Table 2,

*We put the table there to show how the participant’s response looks like regarding each item. So, people can have a look at them and see the responses on each item. But, as you mentioned we can’t explain each item one by one. Our aim was not also primarily to explain responses on each item. Rather, it was to show the overall practice of nurses regarding prevention of SSI and the factors associated with it.*

5) Discussion – please discuss further your results stated in Table 2
Overall – please check the typo [e.g., p value (small “p”), and please avoid “And” as opening the first sentence]
 *As you said it is difficult to discus point by point all of the items present in table 2. Our aim was not also primarily to explain responses on each item. Rather, it was to give an explanation on the overall practice of nurses regarding prevention of SSI. That means on the 2 categorical outcomes “Good and poor “or proper and not proper. So we have tried to discuss the findings in this context. Therefore, Table 2 was put here to give an impression on each item to readers on the practice of nurses regarding SSI prevention.*

*Regarding, p value we decided that, since a p vaue <0.05 means chance is unlikely, the association is significant. But, small p value doesn’t tell us the weakness or strongness of the association. So, we put the significance level at p value <0.05 for all associated variables.*

*“And” is removed from opening statement and modified.*

*Thank you very much.*

*We have considered all the genuine points you raised and made corrections accordingly.*
